# Enzymatically Modified Starch Favorably Modulated Intestinal Transit Time and Hindgut Fermentation in Growing Pigs

**DOI:** 10.1371/journal.pone.0167784

**Published:** 2016-12-09

**Authors:** M. A. Newman, Q. Zebeli, K. Velde, D. Grüll, T. Molnar, W. Kandler, B. U. Metzler-Zebeli

**Affiliations:** 1 Institute of Animal Nutrition and Functional Plant Compounds, University of Veterinary Medicine Vienna, Vienna, Austria; 2 Research Cluster “Animal Gut Health”, Department for Farm Animals and Veterinary Public Health, University of Veterinary Medicine Vienna, Vienna, Austria; 3 Equine University Clinic, Department for Companion Animals and Horses, University of Veterinary Medicine, Vienna, Austria; 4 Agrana Research & Innovation Center GmbH, Tulln, Austria; 5 Center for Analytical Chemistry, Department of Agrobiotechnology, IFA-Tulln, University of Natural Resources and Life Sciences, Vienna, Austria; 6 University Clinic for Swine, University of Veterinary Medicine, Vienna, Austria; National Institute for Agronomic Research, FRANCE

## Abstract

Aside from being used as stabilizing agents in many processed foods, chemically modified starches may act as functional dietary ingredients. Therefore, development of chemically modified starches that are less digestible in the upper intestinal segments and promote fermentation in the hindgut receives considerable attention. This study aimed to investigate the impact of an enzymatically modified starch (EMS) on nutrient flow, passage rate, and bacterial activity at ileal and post-ileal level. Eight ileal-cannulated growing pigs were fed 2 diets containing 72% purified starch (EMS or waxy cornstarch as control) in a cross-over design for 10 d, followed by a 4-d collection of feces and 2-d collection of ileal digesta. On d 17, solid and liquid phase markers were added to the diet to determine ileal digesta flow for 8 h after feeding. Reduced small intestinal digestion after the consumption of the EMS diet was indicated by a 10%-increase in ileal flow and fecal excretion of dry matter and energy compared to the control diet (*P*<0.05). Moreover, EMS feeding reduced ileal transit time of both liquid and solid fractions compared to the control diet (*P*<0.05). The greater substrate flow to the large intestine with the EMS diet increased the concentrations of total and individual short-chain fatty acids (SCFA) in feces (*P*<0.05). Total bacterial 16S rRNA gene abundance was not affected by diet, whereas the relative abundance of the *Lactobacillus* group decreased (*P*<0.01) by 50% and of *Enterobacteriaceae* tended (*P*<0.1) to increase by 20% in ileal digesta with the EMS diet compared to the control diet. In conclusion, EMS appears to resemble a slowly digestible starch by reducing intestinal transit and increasing SCFA in the distal large intestine.

## Introduction

Chemically modified starches (CMS) are common ingredients in processed food items to improve their rheological characteristics and texture [1,2]. Consequently, the daily intake of CMS increases as the consumption of processed foods expands [1,2]. If CMS has further health-promoting properties and hence act as functional dietary ingredients has been less well explored than for naturally occurring starch types [1, 3,4]. Moreover, CMS has received considerable attention lately because the chemical modification of the starch molecule allows for a more exact and defined starch digestibility than predictable for many naturally occurring starches [3, 5–8]. Today’s nutrition contains relatively high proportions of rapidly digestible starches that are typically consumed in the form of processed foods such as breads, cakes, and noodles. There is considerable concern that these rapidly digestible starches may contribute to chronic health disorders, such as insulin resistance and obesity [3]. Therefore, nutritionists have propagated to increase the consumption of slowly digestible or resistant starches (RS) in order to control the glucose release in the small intestine and thus to dilute the digestible energy content of the diet [9]. Slowly digestible starch results in a drawn-out increase in blood glucose, whereas RS completely resists digestion by mammalian enzymes [10]. Without modifying the texture, flavor and appearance of food items, CMS, if less digestible for the host, may be used as an agent to dilute the rapidly digestible carbohydrate intake in humans, but their efficacy needs to be established first. An increase in available substrate at the terminal ileum can result in modulation of the intestinal microbial community and its metabolic activity [7, 11, 12]. Increased hindgut fermentation has been linked to certain health benefits in relation to satiety, insulin secretion, obesity and modulation of the mucosal gene expression response [3, 13, 14]. The latter benefits have attracted the interest in developing foods with elevated concentrations of less digestible starches or RS for incorporation into human diet, and lately, also into pig diets [3, 15].

Recent research on CMS indicated that systemic and intestinal effects may largely diverge from those reported for naturally occurring starches, emphasizing the need to test each CMS separately. As such, we could recently show for an enzymatically modified starch product (EMS) clear differences in its effects on the cecal microbiota [7] and blood metabolites [8] in comparison to reported effects for RS of type 2 and 3 in pigs [13, 16]. Likewise, differing modulatory ability of high-amylose cornstarch and CMS products on the fecal bacterial microbiota in humans and lipid metabolism in knock-out mice models were reported [5, 6]. Evaluation of the effects published for CMS demonstrated a considerable dearth of knowledge regarding CMS effects on passage rate and the bacterial microbiota in the upper intestinal tract. Since pigs have a high similarity in digestive physiology and metabolic responses to humans, observations made in pigs may be applicable for humans [17, 18]. Moreover, using the pig as animal model allows the more complex nature of collecting ileal digesta samples which is hardly achievable from healthy human volunteers.

In considering our earlier findings on large intestinal and systemic effects for EMS [7, 8], we hypothesized a modulated small intestinal starch digestion, passage rate and bacterial starch utilization in the upper digestive tract with consequences for bacterial abundances and metabolite profiles in the large intestine when incorporating EMS as the major carbohydrate source in pig diets. In using the starch component as the sole carbohydrate source, we avoided the interference of other complex carbohydrates except the test starch on our target parameters. We used an ileal cannulation model to differentiate the nutrient disappearance prior to the terminal ileum and throughout the large intestine in order to complete our understanding about the functional abilities of EMS. Therefore, our objective was to determine the effects of enzymatically modified starch (EMS) on ileal flow, post-ileal disappearance, and fecal excretion of nutrients as well as on ileal and fecal gut microbiota and fermentation metabolites in growing pigs.

## Materials and Methods

### Ethical statement

All procedures involving animal handling and treatment were approved by the institutional ethics committee of the University of Veterinary Medicine and the national authority according to paragraph 8 of Law for Animal Experiments, Tierversuchsgesetz–TVG (GZ 68.205/0051-II/3b/2013).

### Animals, housing, and experimental design

Eight crossbred castrated male growing pigs ([Landrace × Large White] × Piétrain; initial BW = 25.9 ± 0.85 kg) from the research pig farm (University of Veterinary Medicine Vienna) were used in the present study. Pigs were surgically fitted with a simple T-cannula (tubus: 9 cm, foot: 9 cm, inner diameter: 2.0 cm, outer diameter: 2.3 cm; LKT—Laboratorium für Kunststofftechnik GmbH, Vienna, Austria), inserted at the terminal ileum to allow for collection of ileal digesta [19, 20]. One week prior to surgery, pigs were moved into 1.0 × 1.2 m individual metabolism pens for adaptation to the new environment, where they were housed for the duration of the experiment. Pens were comprised of Plexiglas walls to allow visual contact and completely slatted plastic flooring. Additionally, each pen was equipped with a single-space feeder and a nipple drinker for *ad libitum* access to demineralized water. The experiment was conducted in an environmentally controlled room (21 ± 1°C). Health was monitored daily and illness was determined by appetite, lethargy, behavior, and/or cannula function. Post-surgery, the skin around the cannula was cleaned with lukewarm water several times daily, treated with a skin-protecting paste (Stomahesive Paste, Convatec, Princeton, UK), and foam material was placed between the retaining ring and the skin to absorb leaking digesta to prevent erythema [21]. Pigs received new empty plastic water bottles as chewing material every day.

During the 9-d recovery period after surgery, pigs consumed a commercial grower diet (metabolizable energy (ME) = 3.19 Mcal/kg; crude protein (CP) = 16.8%, as-fed basis). Feed amounts were gradually increased after surgery until reaching pre-surgery levels. Following recovery, pigs were randomly allotted to 1 of 2 dietary treatments according to a complete crossover design with two 17-d replicate periods. Each replicate period consisted of 10 d acclimation to diets, followed by 4 d of fecal collection, and then 3 d of ileal digesta collection. Four pigs were allotted per diet in each of the 2 replicate periods, which provided a total of 8 observations per dietary treatment.

After completion of the experiment, pigs were anesthetized (Narketan, 10 mL/kg body weight; Ketamine HCl; Vétoquinol AG, Ittigen, Austria; and Stresnil, 3 mL/kg body weight; Azaperone; Biokema SA, Crissier, Switzerland) and euthanized by intracardiac injection of T61 (10 mL/kg; Embutramide; MSD Animal Health, Vienna, Austria).

### Diets

The 2 semi-purified experimental diets were based on purified cornstarch, casein, lignocellulose (FibreCell M1; agromed Austria GmbH, Kremsmünster, Austria), and rapeseed oil (Table 1). Vitamins and minerals were added to meet or exceed requirements (NRC, 2012), and titanium dioxide (TiO_2_; 0.3%) was included as an indigestible marker. Diets were formulated to be identical, with the exception of their starch component. The starch utilized in the control diet was a rapidly digestible waxy cornstarch (Agrana Research and Innovation Center GmbH (ARIC), Tulln, Austria), whereas the starch utilized in the test diet was an enzymatically modified waxy cornstarch (EMS; ARIC). The enzymatic treatment [Enzyme E.C.2.4.1.18: 1,4-α-D-glucan:1,4-α-D-glucan 6-α-D-(1,4-α-D-glucano)-transferase] increased the branching of the starch molecule, which resulted in the amylopectin fraction of the EMS containing more α-1,6 glycosydic bonds (8%) compared to the unmodified waxy cornstarch (4%) [7, 8]. Since the enzymatic treatment also reduced molecular weight, the dextrose equivalent, used as a measure of the number of reducing sugars present relative to glucose, of the EMS was quantified. This was determined to be below 1, indicating that virtually no low-molecular-weight sugars were present. Additionally, mono- and disaccharides were absent to the greatest extent possible. The EMS was further described as a free-flowing, slightly hygroscopic powder. The analyzed nutrient concentration of the diets is presented in Table 1.

**Table 1:** Ingredient and analyzed nutrient composition of experimental diets.

Item	Control diet	EMS diet
Ingredient composition, %		
Waxy cornstarch	72.10	0.00
Enzymatically modified starch^1^	0.00	72.10
Casein	18.00	18.00
Lignocellulose^2^	4.00	4.00
Rapeseed oil	1.00	1.00
Monocalcium phosphate	4.00	4.00
Vitamin-mineral premix^3^	0.60	0.60
Titanium dioxide	0.30	0.30
Analyzed nutrient composition (g/kg DM unless otherwise stated)		
Gross energy (MJ/kg)	16.78	16.95
Dry matter	941	938
Crude protein	166	167
Total starch	689	665
Calcium	7.7	7.9
Phosphorus	4.7	4.8

Nutrient composition presented on a dry matter basis.

^1^ EMS, enzymatically-modified starch (Agenanova; AGRANA, Tulln, Austria).

^2^FibreCell (agromed Austria GmbH, Austria).

^3^Provided per kilogram of complete diet (GARANT GmbH, Austria): 16,000 IU of vitamin A, 2,000 IU of vitamin D_3_, 125 mg of vitamin E, 2.0 mg of vitamin B_1_, 6.0 mg of vitamin B_2_, 3.0 mg of vitamin B_6_, 0.03 mg of vitamin B_12_, 3.0 mg of vitamin K_3_, 30 mg of niacin, 15.0 mg of pantothenic acid, 900 mg of choline chloride, 0.15 mg of biotin, 1.5 mg of folic acid, 200 mg of vitamin C; 4.6 g of Ca, 2.3 g as digestible P, 2.4 g as Na, 2.0 g of Cl, 3.2 g K, 1.0 g Mg; 50 mg of Mn (as MnO); 100 mg of Zn (as ZnSO_4_); 120 mg of Fe (as FeSO_4_), 15.6 mg of Cu (as CuSO_4_), 0.5 mg of Se (as Na_2_SeO_3_), 1.9 mg of I (as Ca(IO_3_)_2_), 3 g TiO_2._

Experimental diets were fed at approximately 3.0 times the estimated energy required for maintenance [22] based on the average weight of pigs at the start of each replicate period. Daily feed allowance was divided into 2 equal meals that were fed at 0800 and 1600 h as a mash and mixed with water at a ratio of about 2:1.

### Sample collection and digesta flow marker application

Pens were scraped and washed daily, and fresh fecal samples were collected from slatted flooring and tray beneath the cage on d 11 to 14 via grab sampling. Subsamples of freshly defecated feces were immediately frozen at -80°C for later bacterial analysis, and remaining samples were stored at -20°C for all other analyses. Ileal digesta samples were collected on d 15 and 16 from 0800 to 1800 h into 10 × 4 cm plastic bags that were attached to the barrel of each cannula using rubber bands. Each bag contained 4 mL of 2.5 M formic acid to minimize bacterial fermentation. Bags were removed when they were full with digesta, or at least every 30 min. At 1100 and 1400 h, ileal digesta samples were collected without formic acid for bacterial microbiota, short-chain fatty acids (SCFA), and lactate analyses. Subsamples for bacterial microbiota were immediately frozen at -80°C, and subsamples for SCFA and lactate were stored at 4°C until digesta samples from both time points were obtained. Then, digesta was pooled by pig, homogenized, and frozen at -20°C for later analysis.

On d 17 of each experimental run, 40 mL of liquid marker (chromium-ethylenediaminetetraacetic acid; Cr-EDTA) and 1 g of solid marker (ytterbium oxide; Yb_2_O_3_) were mixed into the morning meal and offered to pigs as a pulse dose. Ileal digesta samples were collected postprandially at 0, 30, 90, 120, 180, 240, 300, 360, 420, and 480 min [23, 24]. As ileal digesta flow can be irregular, actual collection times were recorded. If no sample could be collected within 15 min of the predetermined collection times, cannulae were closed until the subsequent collection time.

### Chemical analysis

At the conclusion of the experiment, fecal and ileal digesta subsamples for proximate nutrient analysis were lyophilized (Gamma 2–20, Martin Christ Gefriertrocknungsanlagen GmbH, Osterode am Harz, Germany) and ground through a 0.5 mm screen (GRINDOMIX GM200, Retsch GmbH, Haan, Germany) prior to chemical analyses. Diet samples were also homogenized and ground prior to analyses.

Feed, lyophilized feces, and ileal digesta were analyzed in duplicate for dry matter, protein, and ash [25]. Gross energy content in feed, feces, and ileal digesta was determined using an isoperibolic bomb calorimeter (C200, IKA^®^-Werke GmbH & Co. KG, Staufen, Germany), with benzoic acid as the standard used to calibrate the instrument. Titanium dioxide was measured in feed, feces, and ileal digesta according to the method described by Khol-Parisini et al. [26]; absorption was measured at 405 nm using a spectrophotometer (Hitachi U-3000, Metrohm INULA GmbH, Vienna, Austria). Total starch content in feed, lyophilized ileal digesta and fecal samples as well as d- and l-lactate concentrations in fresh ileal digesta and fecal samples were determined using commercially available kits (Megazyme K-TSTA and K-DLATE, Wicklow, Ireland). SCFA concentrations in fresh ileal digesta and fecal samples were analyzed using gas chromatography as recently described [7].

Chromium and Yb was determined by inductively coupled plasma mass spectrometry. A double-focusing sector field instrument, Finnigan ELEMENT2 (Thermo Electron Corporation, Bremen, Germany) equipped with a CETAC ASX-520 autosampler (CETAC Technologies, Omaha, NE, USA) was used. Samples of 50 mg lyophilized ileal digesta were weighed into 10 mL glass tubes and digested with 2 ml 65% nitric acid (60 min at 95°C). The solutions were transferred into cleaned and pre-weighted 50 mL tubes of polypropylene, filled up to 50 g on a laboratory balance and shaken. After sedimentation, the solutions were diluted 1:100 with 0.5% (v/v) nitric acid. As internal standards, 20 μg/L Sc, 10 μg/L In and 10 μg/L Tl were added. The following nuclides were measured in medium-resolution mode, Rs = 4000, 10% valley definition ^31^P, ^44^Ca, ^45^Sc, ^52^Cr, ^173^Yb and ^205^Tl. Quantitative analysis of the samples was performed by external calibration.

### DNA extraction and quantitative PCR

Total DNA extraction and purification of DNA were performed essentially as described in Metzler-Zebeli et al. [7]. Briefly, DNA were extracted from 250 mg of ileal digesta and fecal samples using a PowerSoil DNA isolation kit (MoBio Laboratories Inc., Carlsbad, CA). An additional heating step at 70°C for 10 min was introduced between mixing of the sample with the commercial buffer C1 and the bead-beating step to ensure proper lysis of bacteria. Eluted DNA was purified using the NucleoSpin Tissue Kit (Machery Nagel, Düren, Germany). The DNA concentration was determined by a Qubit 2.0 Fluorometer (Life Technologies, Carlsbad, CA, USA) using the Qubit dsDNA HS Assay Kit (Life Technologies) and ranged from 2 to 55 ng/μL. To achieve similar DNA concentrations across samples, DNA extract volumes were adjusted using Tris buffer.

Quantitative PCR (qPCR) assays were performed with the Stratagene Mx3000P QPCR System (Agilent Technologies, Santa Clara, CA), using Brilliant II SYBR Green QPCR Low ROX master mix (Agilent Technologies), forward and reverse primers (62.5 pmol/μL) and 1 μL of genomic DNA in a final volume of 25 μL, to quantify the abundance of 16S rRNA genes of total bacteria and target bacterial groups (i.e. *Lactobacillus* group, *Bifidobacterium* spp. *Enterobacteriaceae*, *Escherichia coli*, *Clostridium* cluster I, IV and XIV and *Bacteroides-Prevotella-Porphyromonas*), whereby targeted bacterial groups comprised starch-degrading and proteolytic species [27, 28]. All qPCR amplifications consisted of initial denaturation at 95°C for 10 min, followed by 40 cycles of 95°C for 15 seconds, annealing at 60°C for 30 seconds (except for universal primers (61°C), *Lactobacillus* group (62°C) and *Enterobacteriaceae* (63°C) annealing temperature was 60°C), and elongation at 72°C for 30 seconds. Fluorescence was measured at the last step of each cycle. Standards and samples were run on the same plate in duplicate. Negative controls without template DNA were included in triplicate. Melting curve analysis from 55 to 95°C and horizontal gel electrophoresis were performed to determine the specificity of the amplification [7].

For quantification of bacterial 16S rRNA gene copies, standards were prepared via serial dilutions (10^7^ to 10^3^ molecules/μL) of the purified and quantified PCR products generated by standard PCR and genomic DNA from pig intestinal digesta [7, 27]. Amplification efficiencies were calculated according to the following equation: *E* = 10^−1/slope^ and ranged from 1.90 to 2.01. Linear relationships between quantification cycle (Cq) and log of DNA concentration were observed for each primer pair (*R*^*2*^ = 0.997 to 1.000). Gene copy numbers of total bacteria and target bacterial groups were determined by relating the C_q_ values to standard curves and by considering dilution volume of extracted DNA, DNA amount subjected to analysis, and the weight of the sample subjected to DNA extraction. The percentage of each target bacterial group was obtained by dividing the gene copies of the 16S rRNA gene of the target group by the 16S rRNA genes amplified with the universal bacterial primer set [7].

### Calculations

The amount of dry matter, protein, ash, starch (g/kg dry matter intake (DMI)), and GE (Mcal/kg DMI) remaining at the terminal ileum and excreted in feces were calculated using the following equations:

$$\text{Amount remaining at terminal ileum }(\text{g or Mcal}/\text{kg DMI})\;=\;[\text{Concentration of dietary constituent in digesta }\times \;({\text{TiO}}_{2}\text{ in diet }/{\text{ Tio}}_{2}\text{ in digesta})].$$

$$\text{Fecal excretion of dietary constituents }(\text{g or Mcal}/\text{kg DMI})=[\text{Concentration of dietary constituent in feces }\times ({\text{TiO}}_{2}\text{ in diet }/\;{\text{TiO}}_{2}\text{ in feces})].$$

Disappearance of dry matter, protein, ash, starch (g/kg DMI), and GE (Mcal/kg DMI) in the large intestine was calculated using the following equation:

Post-ileal disappearance (g or Mcal/kg DMI) = amount remaining at terminal ileum – amount excreted in feces.

Post-ileal disappearance (g or Mcal/kg DMI) = amount remaining at terminal ileum–amount excreted in feces.

Total SCFA and lactate contents produced (μmol/g DMI) in ileal digesta or feces were calculated as ileal digesta or feces total SCFA content (μmol/g DMI) = (SCFA concentration in mmol in ileal digesta or feces × total amount of ileal digesta or feces in g)/total daily DMI in g.

The excretion curves of ileal markers including Yb as a solid marker and Cr as a liquid marker were fitted individually for each pig using an age-dependent kinetics model with time delay [29], implemented in the NLIN procedure (iterative Marquardt method) of SAS (Version 9.3, SAS Inst. Inc., Cary, NC). The time delay indicates the time lapse between pulse dosing and the first appearance of markers in the ileal digesta (or transit time due to displacement flow of digesta in minutes). The time lapse between pulse dosing and the reach of peak ileal marker excretion was considered as the time of peak flow (minutes).

### Statistical analysis

This experiment was designed as a complete crossover design with 2 dietary treatments and 2 replicate runs. To compare differences between diets, data were subjected to ANOVA using the MIXED procedure of SAS (Version 9.3, SAS Inst. Inc., Cary, NC). The fixed effect of diet and the random effect of pig nested within experimental run were included in the main model, considering the individual pig as experimental unit. Degrees of freedom were approximated using Kenward-Rogers method (ddfm = kr). Differences were reported as least-square means and were considered significant if *P* < 0.05 and were described as tendencies if 0.05 ≤ *P* < 0.10.

## Results

### Animals and diets

All animals remained healthy for the duration of the experiment. Furthermore, analyzed nutrient composition showed very little difference between dietary treatments (Table 1); hence the two diets contained equal amounts of starch.

### Intestinal nutrient flow

Pigs completely ate the offered feed amount, resulting in similar daily dry matter, ash, starch, protein, and energy intake among diets (Table 2). Diets caused characteristic differences in ileal nutrient flow and post-ileal nutrient disappearance. Though no differences in ileal flow of ash and protein were observed, pigs fed the EMS diet had (*P*<0.05) about 10%-greater flow of dry matter, organic matter and energy at the terminal ileum, and 8 to 10%-higher (*P*<0.05) concentrations in the feces compared to pigs fed the control diet, respectively. Post-ileal dry matter and energy disappearance were not affected by the EMS diet. However, apparent ileal flow and post-ileal disappearance of starch were reduced (*P*<0.05) by 23% and 25%, respectively, in pigs fed the EMS diet compared to those fed the control diet (Table 2).

**Table 2:** Intake and nutrient flow along gastrointestinal tract in pigs fed control and enzymatically-modified starch (EMS) diets.

Item^1^^,^^2^^,^^3^^,^^4^	Control diet	EMS^5^ diet	SEM	*P*-value
Intake				
Dry matter, g/d	1124	1138	—	—
Organic matter, g/d	1080	1094	—	—
Ash, g/d	774	756	—	—
Starch, g/d	188	189	—	—
Protein, g/d	45	44	—	—
Gross energy, MJ/d	18.8	19.0	—	—
Dry matter, g/kg DMI				
Remaining at TI	174	194	5.62	0.029
Postileal disappearance	35	44	6.11	0.329
Excreted in feces	135	150	3.40	0.008
Organic matter, g/kg DMI^6^				
Remaining at TI	145	160	4.34	0.029
Postileal disappearance	31	37	5.73	0.489
Excreted in feces	110	123	2.79	0.006
Starch, g/kg DMI				
Remaining at TI	62	47	2.28	<0.001
Postileal disappearance	61	45	3.35	<0.001
Excreted in faeces	1	1	0.08	0.336
Protein, g/kg DMI				
Remaining at TI	26	29	1.93	0.280
Postileal disappearance	16	18	1.83	0.440
Excreted in feces	10	12	0.51	0.080
Ash, g/kg DMI				
Remaining at TI	30	34	2.10	0.171
Postileal disappearance	4	7	1.01	0.042
Excreted in feces	25	27	1.34	0.281
GE, MJ/kg DMI				
Remaining at TI	2.72	2.99	0.08	0.035
Postileal disappearance	0.52	0.59	0.13	0.684
Excreted in feces	2.15	2.41	0.10	0.020

All values presented as least square means ± SEM; n = 8 per dietary treatment. DMI, dry matter intake. TI, terminal ileum.

^1^Pigs ate similar amounts of feed (on as fed basis); average amounts of both periods.

^2^Amount remaining at terminal ileum = [component in digesta × (TiO_2_ in diet / TiO_2_ in digesta)].

^3^Postileal disappearance = [amount remaining at terminal ileum–amount excreted in feces].

^4^Amount excreted in faeces = [component in feces × (TiO_2_ in diet / TiO_2_ in feces)].

^5^Agenanova. (AGRANA, Tulln, Austria).

^6^Organic matter = dry matter–ash.

### Passage rate

Both liquid (Cr-EDTA) and solid (Yb_2_O_3_) phase markers were added to the morning meal on experimental d 17 to better understand rate of passage through the stomach and small intestine. Regardless of diet, the first solid phase marker appeared approximately 80 min after the first liquid phase marker (Table 3). Time of peak marker excretion was close to 45 min later for the solid phase compared to the liquid, again, irrespective of the diet. In pigs fed the EMS diet, time of peak passage for both the liquid and solid phase markers through the terminal ileum was approximately 2 h later (*P*<0.05 and *P*<0.10, respectively) than it was for control-fed pigs. Additionally, there was a time delay of approximately 1 h (*P*<0.05) before the first appearance of the solid phase marker at the terminal ileum in pigs fed the EMS diet compared to control. Differences between peak time of marker excretion and time delay for liquid and solid phase passage rate markers were similar between the two diets.

**Table 3:** Digesta passage variables at the terminal ileum of pigs fed control and enzymatically-modified starch (EMS) diets.

Item	Control diet	EMS^1^ diet	SEM	*P*-value
Liquid phase (min)				
Time delay	209	278	33.3	0.175
Time of peak flow	269	376	27.4	0.019
Difference^2^	59	98	25.9	0.314
Solid phase (min)				
Time delay	288	358	22.3	0.049
Time of peak flow	306	424	42.0	0.075
Difference^2^	91	91	21.8	0.996

All values presented as least square means ± SEM; n = 8 per dietary treatment.

^1^Agenanova (AGRANA, Tulln, Austria).

^2^Difference between time of peak flow and time delay.

### Fermentation metabolites and bacterial abundances

**Fig 1:**
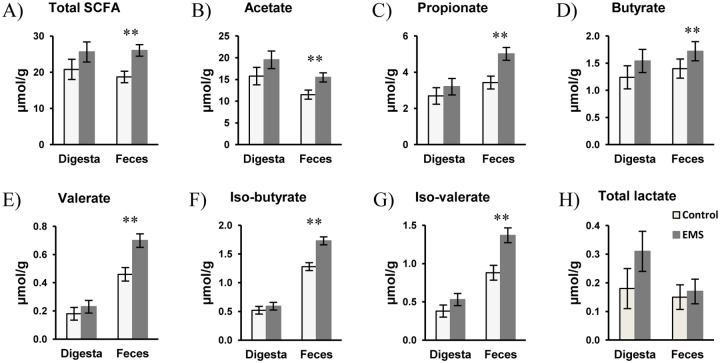
Total SCFAs (A), acetate (B), propionate (C), butyrate (D), valerate (E), isobutyrate (F), isovalerate (G), and total lactate (H) concentrations in ileal digesta and feces of pigs fed the control or enzymatically modified starch (EMS) diet. Values are presented as least square means ± SEM; n = 8 per dietary treatment. **Control diet and EMS diet differ within the ileal digesta or feces, *P*<0.05.

Dry matter content and pH of ileal digesta and feces were similar between diets (Table 4). Lactate concentrations in ileal digesta and feces (on a wet basis) were equal between diets (Fig 1). Also, no differences in SCFA concentrations were observed in ileal digesta, whereas the EMS diet increased (*P*<0.05) the total SCFA concentration in feces (on a wet basis) by nearly 40% compared to the control diet (Fig 1). Although individual SCFA were present in feces at greater concentrations with the EMS diet (*P*<0.05), molar SCFA proportions remained similar when compared to the control diet. Despite differing ileal dry matter flow, expression of ileal and fecal lactate and SCFA as total produced calculated on the basis of dry matter flow and nutrient intake showed similar total lactate and total SCFA content in ileal digesta (per g of DM fed; *P*>0.1; Table 4). At fecal level, when results were expressed as total lactate and total SCFA content (per g of DM fed) the enhancing effect of EMS on fermentation became more obvious (*P*<0.01) compared to the control diet.

**Table 4:** Characteristics of digesta and total lactate and short-chain fatty acid (SCFA) output at the terminal ileum and feces of pigs fed control and enzymatically-modified starch (EMS) diets.

Item	Control diet	EMS^1^ diet	SEM	*P*-value
Ileal digesta				
Dry matter content (g/kg)	80	94	1.1	0.387
pH	8.8	8.6	0.11	0.185
Total lactate (μmol/g DMI)^2^	0.03	0.06	0.015	0.129
Total SCFA (μmol/g DMI)^2^	3.10	4.34	0.492	0.101
Feces				
Dry matter content (g/kg)	551	528	25.0	0.545
pH	8.4	8.6	0.16	0.429
Total lactate (μmol/g DMI)^2^	0.02	0.04	0.012	0.241
Total SCFA (μmol/g DMI)^2^	2.30	3.56	0.252	0.004

All values presented as least square means ± SEM; n = 8 per dietary treatment. DMI, dry matter intake.

^1^Agenanova (AGRANA, Tulln, Austria).

^2^Ileal digesta or feces total lactate and SCFA content produced (μmol/g DM fed) = [(lactate or SCFA concentration in μmol in ileal digesta or feces × total amount of ileal digesta or feces in g)/total daily dry matter intake in g].

Dietary inclusion of EMS did not affect (*P*>0.10) total bacterial gene copies in ileal digesta or feces (Table 5), but did alter bacterial populations by largely decreasing (*P*<0.01) the relative abundance of the *Lactobacillus* group by about 50% in ileal digesta compared to the control starch. Concurrently, pigs fed the EMS diet tended (*P*<0.10) to have 20%-units more *Enterobacteriaceae* in ileal digesta compared to pigs fed the control diet, whereby the ileal abundance of *Escherichia coli* was not modified by diet. In feces, differences in the bacterial abundances between diets were no longer traceable in feces. Likewise, bacterial groups including members with amylase and pullulanase activity, such as *Clostridium* cluster IV and XIV as well as *Bacteroides-Prevotella-Porphyromonas*, were not different between diets in ileal digesta and feces.

**Table 5:** Total bacterial abundance and relative bacterial group abundance in ileal digesta and feces of pigs fed control and enzymatically-modified starch (EMS) diets.

Item	Control diet	EMS^1^ diet	SEM	*P*-value
Ileal digesta				
Total bacteria, log_10_ gene copies/g % of total bacteria	9.3	9.3	0.139	0.832
*Lactobacillus* group	77.50	24.89	11.763	0.008
*Clostridium* cluster I	0.31	0.50	0.263	0.617
*Clostridium* cluster IV	0.48	0.40	0.169	0.750
*Clostridium* cluster XIV	0.57	0.58	0.134	0.996
*Bifidobacterium* spp.	8.4×10^−5^	8.8×10^−5^	4×10^−5^	0.940
*Bacteroides-Prevotella-Porphyromonas*	6.67	6.07	1.097	0.707
*Enterobacteriaceae*	14.96	35.40	7.667	0.082
*Escherichia coli*	0.09	0.16	0.038	0.198
Feces				
Total bacteria, log_10_ gene copies/g % of total bacteria	10.3	10.1	0.180	0.572
*Lactobacillus* group	18.69	17.06	6.515	0.863
*Clostridium* cluster I	3.26	11.96	4.188	0.168
*Clostridium* cluster IV	2.72	13.60	5.220	0.166
*Clostridium* cluster XIV	1.60	3.84	1.337	0.261
*Bifidobacterium* spp.	4.2×10^−5^	1.15×10^−4^	4.4×10^−5^	0.265
*Bacteroides-Prevotella-Porphyromonas*	12.12	10.09	1.652	0.402
*Enterobacteriaceae*	2.47	2.91	0.918	0.740
*Escherichia coli*	0.04	0.04	0.015	0.781

All values presented as least square means ± SEM; n = 8 per dietary treatment.

^1^Agenanova (AGRANA, Tulln, Austria).

## Discussion

Aside from their texture-enhancing properties, the specific modification of the starch molecule in CMS may allow for the design of starches with functional abilities. However, information is scarce on intestinal and physiological effects of CMS. The present study provides evidence for EMS to slow down small intestinal digestion and passage rate as well as to increase post-ileal fermentation in growing pigs. These findings may have contributed to the lower feed efficiency in pigs fed the EMS diet in our previous study [7]. Contrary to studies on RS in pigs [16, 30], we did not observe similar benefits on ileal protein digestibility and mineral absorption by feeding the EMS. Nevertheless, their ileal digestibilities were not hindered, which is considered essential to prevent a loss in the nutritive value of the whole diet. These findings demonstrate the need for the investigation of each new CMS.

In general, intestinal digesta bulk and nutrient flow are considered to be key factors influencing digesta passage rate and microbial activity throughout the gastrointestinal tract [16, 31]. A positive effect of EMS on ileal and fecal bulk was absent, whereas the current ileal organic matter and energy flow in pigs fed the EMS diet corresponded to increases in ileal nutrient flow of 10 to 30% when RS levels of more than 50% were included in diets for growing pigs [16, 32]. In these studies, digesta flow directly correlated to the starch content that resisted small intestinal digestion [13, 16, 31]. Results for the ileal starch flow, in turn, were contradictory to those for ileal organic matter and energy flow and were not expected, as *in vitro* digestion indicated reduced starch digestibility (data not shown; AOAC, method 2009.01) [33]. It is clear that EMS is not a classical high-amylose or retrograded starch *per se*. Increasing the amount of α-glucosidic-1,6-linkages should have increased the packaging of the glucose molecules in the EMS, thereby hindering the accessibility for α-amylase. Concurrently, the reduction in the overall molecular weight of the starch molecules due to the enzymatic treatment may have facilitated the digestion of the digestible starch fraction in EMS. This reasoning may explain the lack of moderation in glucose release after EMS ingestion in our jugular-vein catheterized pig model [8] and may also be valid in the present study. Still, this reasoning cannot explain the discrepancies in ileal flow of nutrients; other factors evidently contributed to these findings.

Ileal flow measurements did not account for changes in intestinal passage rate, as digesta was collected and pooled over the entire 10-h period. The delay in ileal excretion of liquid and solid phase passage rate markers of 1 to 2 h, however, indicated reduced gastric emptying and small intestinal passage with the EMS diet, which may explain part of our observation for the ileal starch flow between diets. Increasing the branching should have rendered the EMS more soluble in comparison to the original waxy cornstarch. This, together with reduced starch hydrolysis in the small intestine, likely increased the retention of the liquid and solid phase in the stomach [34]. Slower intestinal passage of the EMS diet may have, therefore, compensated for the reduced α-amylolytic hydrolysis and thus glucose release. These features commonly classify a slowly digestible starch [10]. It might be applicable to categorize the present EMS as such, which may show potential benefit for inclusion in both human and pig diets, as slower gastric and small intestinal passage can promote satiety, thereby decreasing overall food intake in humans and improving welfare in restrictedly fed sows [15, 35].

In general, when feeding a semi-purified diet, the dietary ingredients are highly digestible, except for the cellulose component. Consequently, purified rapidly digestible waxy cornstarch can be reasonably expected to achieve near 100% digestibility prior to the terminal ileum [36]. The intestinal capacity to absorb glucose, however, may become limiting due to negative feedback mechanisms when dietary carbohydrate concentrations exceed 50–60% in the diet [37]; which we exceeded with more than 70% dietary starch content. In assuming that absorptive processes were incomplete until the distal segment of the small intestine in pigs fed the control diet, this may provide some additional justification for the ileal starch flow in pigs fed the control diet. Yet, besides the non-digestible cellulose (43 g/kg DMI) and little protein (26–29 g/kg DMI), starch and degradation products should have comprised a substantial portion of the 145–160 g (per kg DMI) organic matter in ileal digesta based on the current dietary ingredient composition. The longer retention time with the EMS diet and reduced sugar absorption with the control diet can hardly justify for the whole discrepancy between ileal organic matter and starch flow. When subtracting the protein flow and cellulose (per kg DMI) from the organic matter flow to roughly estimate the ileal starch flow, an approximate 10 g more starch (per kg DMI) would have flowed into the large intestine with the EMS diet compared to the control diet. Although the enzymatic-photometric method used in the current study has been applied previously to determine the starch content in ileal digesta and feces of pigs fed semi-purified diets [16, 24, 38], an incomplete recovery of starch in ileal digesta of pigs fed the EMS diet due to possible changes in digesta viscosity cannot be completely ruled out. Also, the total starch content was 20 g less for the EMS diet compared to the control diet, despite similar amounts of purified starches incorporated in both diets. Finally, the difference between the measured amounts of organic matter and the sum of starch, protein and cellulose amounted to 14 and 41 g/kg DMI remaining at the terminal ileum and 46 and 67 g/kg DMI in feces for the control and EMS diets, respectively. In accounting for microbial metabolites in ileal digesta and feces, it is therefore feasible that the assay kit used to detect starch may have incompletely hydrolyzed the starches in digesta and feces as glucose released from the starches is used to estimate the total starch contents. This may have been particularly true for the modified starch as the assay kit were developed for naturally occurring starches.

Microbial activity occurs throughout the gastrointestinal tract [13, 38]. As such, microbial degradation of starch and fermentation metabolites contribute to digestibility coefficients and dry matter flow, respectively. Slower small intestinal passage in pigs fed the EMS diet would have extended the contact time between bacteria and digesta and hence stimulated microbial fermentation. In general, dietary starches, both digestible and resistant, are easily degradable for microbes in the upper part of the digestive tract [13, 16, 32]. Equal SCFA concentrations and pH of ileal digesta supported high fermentability of the two starches, which was inconsistent with our recent findings for large intestinal fermentation [7]. In that study, lower SCFA concentrations in cecal and proximal colonic digesta of pigs fed the EMS diet suggested reduced microbial ability to utilize EMS. This was supported by reduced cecal abundance of bacterial pathways for starch degradation and SCFA generation with the EMS diet using functional metagenome prediction via Kyoto Encyclopedia of Genes and Genomes (KEGG) pathway analysis [7]. The SCFAs detected in digesta only represent the non-absorbed proportion of the total amount of SCFAs produced and it is possible that differences between diets were masked by increased SCFA absorption in the small intestine [39]. Also, differences in gut site and feeding level as well as the ileal cannulation can likely explain some of the inconsistencies between the present and previous study [8]. Though EMS feeding did not impair ileal SCFA and lactate concentrations, ileal abundance of the *Lactobacillus* group—a group containing many amylolytic species—was drastically depressed, which was similar to our observations in cecal digesta and may be related to lower ability of this bacterial group to utilize the EMS [7]. As present total bacterial abundance was similar between diets, results indicated substantial alterations in the community structure. Obviously, the family *Enterobacteriaceae*, but species other than *E*. *coli*, profited and filled part of the niche that opened from the decrease in the *Lactobacillus* group. Current primer sets covered bacterial genera that were typically promoted by RS type 2 and 3 feeding in pigs (e.g., *Bifidobacterium*, *Blautia*, *Parabacteroides*, *Faecalibacterium*, *Ruminococcus*, *Eubacterium* or *Roseburia* [12, 13, 40–42], as well as those previously seen to respond to the EMS in the cecal microbiome [7]. However, other than the *Lactobacillus* group and *Enterobacteriaceae*, none of the studied bacterial groups responded to the EMS, demonstrating that other, non-covered genera adopted the opened niche in the small intestine. In feces, no obvious changes in the investigated bacterial groups could be detected; indicating that, considering the higher fecal SCFA amounts produced in pigs fed the EMS diet, changes may have been more at metabolic level.

Postprandial disappearance of dry matter, energy, protein, and starch can be directly linked to microbial activity. Except for reduced post-ileal starch disappearance with the EMS diet, dry matter and protein disappearance were equal between diets, thereby indicating similar fermentative activity in the large intestine. Overall, enhanced SCFA concentrations in feces of pigs fed the EMS diet suggested a shift in fermentative activity to the distal part of the large intestine. Although RS type 2 and 3 were reported to stimulate fermentation in the proximal parts of the large intestine [13, 32], EMS may be effective to increase fermentation in the distal part of the large intestine. To support this assumption, greater dry matter and protein excretion in feces with the EMS diet may also suggest greater microbial activity in the distal large intestine. Enhancing carbohydrate fermentation in the distal large intestine via dietary modulation is assumed beneficial for the host animal due to the many effects of straight-chain fatty acids on intestinal and systemic health [1, 3, 43, 44]. Increased fermentation may have rendered minerals more soluble in the large intestine leading to the 0.75-fold increase in post-ileal ash disappearance [45] which may be advantageous in fast-growing modern pig lines. Aside from a rise in SCFA concentrations, dietary inclusion of RS type 2 and 3 was associated with higher proportions of propionate, butyrate, and valerate in the large intestines of pigs [13, 16, 32, 46]. In our study, the EMS diet led to about 30 to 40% increase in each individual SCFA in feces but molar proportions remained similar between diets. Although we could not account for the absorbed SCFA proportion by using the current pig model, there may be a certain potential of EMS in the promotion of intestinal health, appetite suppression, or attenuation of lipid or glucose metabolism via the increase in intestinally-generated SCFAs [3, 43, 44]. Particularly, butyrate may support mucosal functioning as the preferred energy source of colonocytes in both humans and pigs, whereas acetate and propionate contribute to systemic energy homeostasis [47]. Due to the greater availability of carbohydrates, natural RS lowered branched-chain fatty acids, as potential harmful fermentation products and markers for protein fermentation [48], in the large intestine of growing pigs [13]. Although branched-chain fatty acids were elevated by the EMS feeding in the present study, the post-ileal disappearance of protein, however, indicated similar protein fermentation in the large intestine. Observed changes in serum lipid metabolome profiles of jugular vein catheterized pigs suggested a certain SCFA-related effect of EMS [8]. Together with a slower ileal passage rate, increased SCFA-activation of mucosal G-protein receptors may lead to an enhanced satiety feeling in growing pigs [15] which may reduce feed-oriented behavior of the pigs and may improve welfare. This may particularly apply for the finishing period when animals are restrictively fed to control the fat percentage of the carcass.

In conclusion, present results suggest that EMS appears to resemble a slowly digestible starch by favorably reducing intestinal transit time and increasing fermentation in the distal large intestine. However, current findings also support the importance to examine physiological effects of each new CMS. Present results further indicate changes in the ileal bacterial community, which is likely in relation to the capability of bacteria to use the EMS. Stimulation of carbohydrate fermentation in the distal large intestine may be a beneficial feature of EMS and may promote host health. Hence, EMS demonstrated a certain potential to be used as a functional ingredient in pig diets, but further research using more complex diets is needed before its incorporation in pig and human diets.
